# OutLyzer: software for extracting low-allele-frequency tumor mutations from sequencing background noise in clinical practice

**DOI:** 10.18632/oncotarget.13103

**Published:** 2016-11-04

**Authors:** Etienne Muller, Nicolas Goardon, Baptiste Brault, Antoine Rousselin, Germain Paimparay, Angelina Legros, Robin Fouillet, Olivia Bruet, Aurore Tranchant, Florian Domin, Chankannira San, Céline Quesnelle, Thierry Frebourg, Agathe Ricou, Sophie Krieger, Dominique Vaur, Laurent Castera

**Affiliations:** ^1^ Department of Cancer Biology and Genetics, CCC François Baclesse, Genomic and Personalized Medicine in Cancer and Neurological Disorders Unit, Caen, France; ^2^ Inserm U1079, Genomic and Personalized Medicine in Cancer and Neurological Disorders Unit, Rouen, France; ^3^ Genetic Department, Rouen University Hospital, Genomic and Personalized Medicine in Cancer and Neurological Disorders Unit, Rouen, France; ^4^ Rouen University, France; ^5^ Caen University, France

**Keywords:** variant-caller, somatic mutation, bioinformatics, oncology, precision medicine

## Abstract

Highlighting tumoral mutations is a key step in oncology for personalizing care. Considering the genetic heterogeneity in a tumor, software used for detecting mutations should clearly distinguish real tumor events of interest that could be predictive markers for personalized medicine from false positives. OutLyzer is a new variant-caller designed for the specific and sensitive detection of mutations for research and diagnostic purposes. It is based on statistic and local evaluation of sequencing background noise to highlight potential true positive variants. 130 previously genotyped patients were sequenced after enrichment by capturing the exons of 22 genes. Sequencing data were analyzed by HaplotypeCaller, LofreqStar, Varscan2 and OutLyzer. OutLyzer had the best sensitivity and specificity with a fixed limit of detection for all tools of 1% for SNVs and 2% for Indels. OutLyzer is a useful tool for detecting mutations of interest in tumors including low allele-frequency mutations, and could be adopted in standard practice for delivering targeted therapies in cancer treatment.

## INTRODUCTION

The advent of Next Generation Sequencing (NGS) during the last decade has been a true revolution both in research and diagnostic laboratories. NGS allows DNA to be read with greater speed and convenience than ever before. The most recent sequencing devices produce the equivalent of several entire genomes in a few hours, while the sequencing of the first human genome took about ten years [1]. NGS is now used daily in clinical laboratories, in many fields of medicine, and particularly in oncology and the diagnosis of cancer predisposition [2]. The molecular typing of tumors in the context of precision medicine could benefit from this technology. For example, somatic mutations in the *EGFR* gene are now commonly sought in lung cancer [3], *KRAS* mutations in colorectal cancer [4] and *BRCA1* and *BRCA2* in ovarian cancer [5]. Thanks to the panel gene sequencing approach, NGS technologies optimize and simplify laboratory processes to the extent that it is today possible to sequence the majority of medical targets of interest in one experiment, regardless of tumor type. When associated with dedicated bioinformatics tools, NGS can explore tumoral heterogeneity and characterize intra-tumoral clonal subpopulations [6]. The identification of sub-clones possibly carrying sensitive or resistance mutations to targeted therapies appears to be a key challenge for patient support in the context of personalized medicine.

The fine characterization of the mutation profile of a tumor with NGS for clinical purposes is a challenge. Diagnostic laboratories therefore have to meet a number of constraints to satisfy the high level of sensitivity and specificity needed for diagnostic tests. Tumoral tissue may include many cell subpopulations, so cells carrying a mutation of interest may be poorly represented in a tumor sample (i.e. low allele-frequency tumor mutations). Moreover, tumor cells can be harvested together with healthy tissue, thereby reducing the number of mutated alleles by dilution. In view of these constraints, a highly sensitive process is required to avoid false negative results. The analysis of sequencing can itself be misleading owing to a PCR reaction bias during sample preparation [7] or to sequencer reading errors [8]. Low level mutations may also be difficult to distinguish from a noise background generated by such technical limitations. Consequently, ensuring a high specificity is critical in diagnostic testing to avoid false positives.

Dedicated bioinformatics tools can help to ensure good sensitivity and specificity. Detection of mutations is a key step in bioinformatics analysis and is performed by variant-calling software. An example of the numerous variant-callers currently available is HaplotypeCaller in the GATK suite [9]. It is a reference in genotyping germline genomes but its sensitivity can dramatically decrease when faced with low level mutations. Others like Varscan2 [10] and LofreqStar [11] have been designed especially for tumor sample analysis and the detection of low level mutations but are efficient mainly for comparing matched healthy and tumor samples. In many diagnostic laboratories a matched healthy sample is not available for analysis owing to ethical considerations, organizational difficulties or legal constraints. Furthermore, even if it were to be available, sequencing would be twice as expansive owing to the need to sequence two different samples for the same patient.

Here we present OutLyzer, a new variant-caller which was validated in a local diagnostic setting to fit ISO15189 quality requirements. It has been designed for non-matched tumoral sample analysis and it is based on statistic and local evaluation of sequencing background noise. It was validated by analyzing paired-ends Illumina data from the targeted resequencing of a gene panel enriched by capture from colorectal, lung, ovarian and breast cancer paraffin-embedded tumors already genotyped during initial diagnostic of cancer. Its analytic performances were compared to those of Varscan2, LofreqStar and also to the well-known HaplotypeCaller (Pubmed: 2222 citations). It produces a powerful, simple and comprehensive analysis with an assessment of sensitivity limits for use in routine practice.

## RESULTS

After sequencing, targeted regions were covered with an average depth of 2111× and 99.46% of nucleotides were covered with a depth > 200×. The 130 samples were analyzed by four different variant-callers, including OutLyzer, to highlight both Single Nucleotide Variations (SNVs) and Insertion-Deletion (Indels) events.

**Figure 1:**
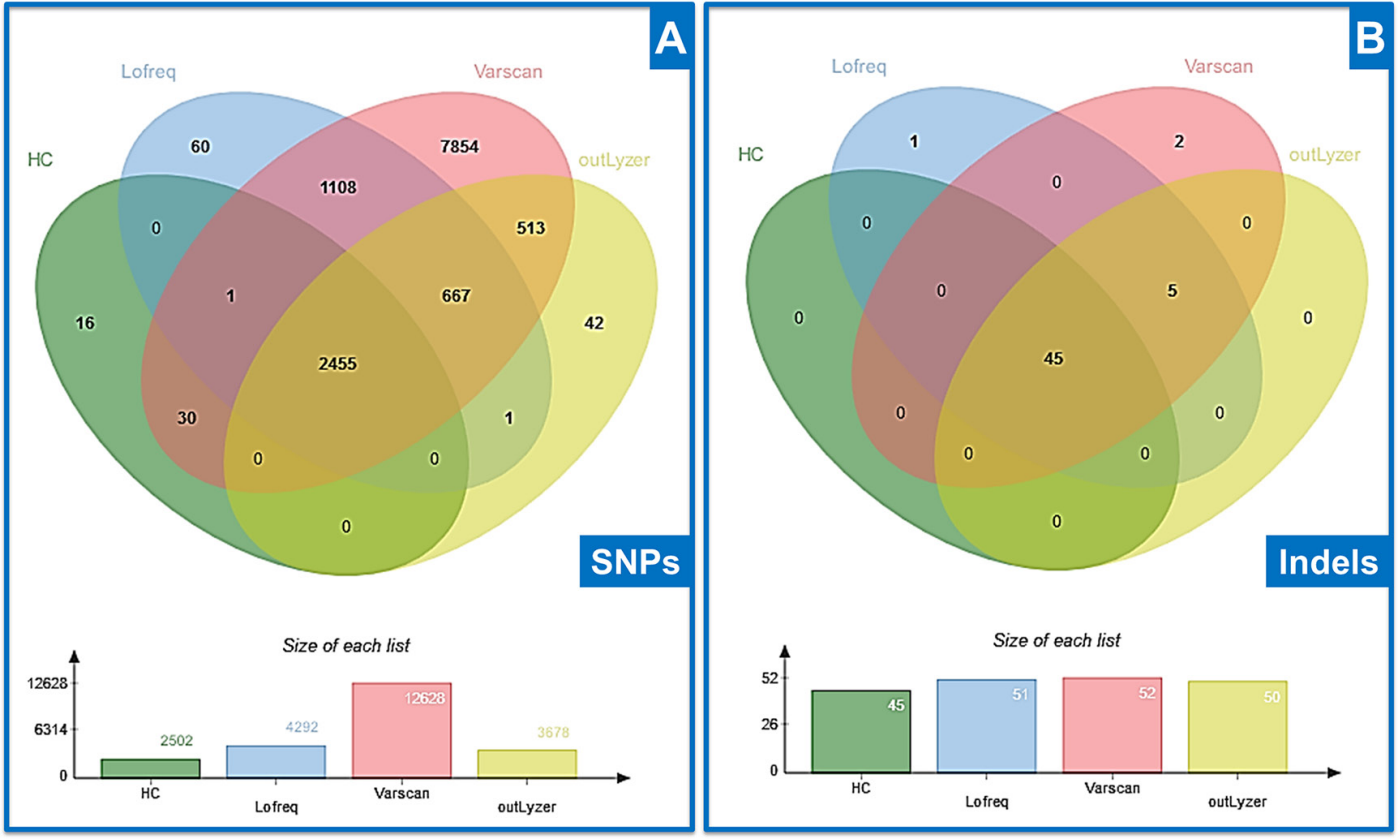
Venn diagram of mutations found by each variant-caller tested In both figures, HC = HaplotypeCaller (**A**) Comparison of SNVs identified (**B**) Comparison of Indels identified.

A total of 12747 SNVs with an allele ratio higher than 1% was identified on coding regions (Figure 1A) and 53 indels with an allele ratio higher than 2% (Figure 1B). SNVs and Indels were processed in two separate benchmark analyses. Regarding SNVs, most mutations detected by all variant-callers were from a probable germline origin with an allele ratio around 50 (heterozygous) or 100 % (homozygous). Among the 30 SNVs detected by both HaplotypeCaller and Varscan, 28 represented one same recurrent event located in an area with mapping issues associated with poor quality metrics. The 16 SNVs detected only by HaplotypeCaller also had a low Phred Score with mapping issues, just like the 60 SNVs, corresponding to 10 unique variants detected by Lofreq alone. Other SNVs found by OutLyzer only, Lofreq and Varscan, OutLyzer and Varscan, or Lofreq and Varscan and OutLyzer together were low allele-ratio events with good quality metrics. SNV found by Varscan only showed lower quality metrics and some were highly recurrent events between samples (190 variants for 5 unique variants).

To enhance the clarity of indel analysis, comparative data were firstly cleaned manually to remove two recurrent false-positives with a low allele ratio (detected by all variant-callers) that were induced by mapping issues and were present in most patients. All the expected events in this dataset were detected by all variant-callers, so their levels of performance were similar (Figure 1B). The 5 indels detected by all of them except HaplotypeCaller were mutations recovered with a low allele ratio and probably of somatic origin. Among the 2 indels identified only by Varscan, one was an artefact caused by a homozygous deletion on the adjacent nucleotide, and the other was a low allele-ratio deletion also identified by OutLyzer but below the limit fixed at 2%. The last indel identified only by Lofreq was also a low allele-ratio deletion supported by only 4 reads. Sensitivity and specificity were then calculated to evaluate and compare performances of each variant-caller.

**Figure 2:**
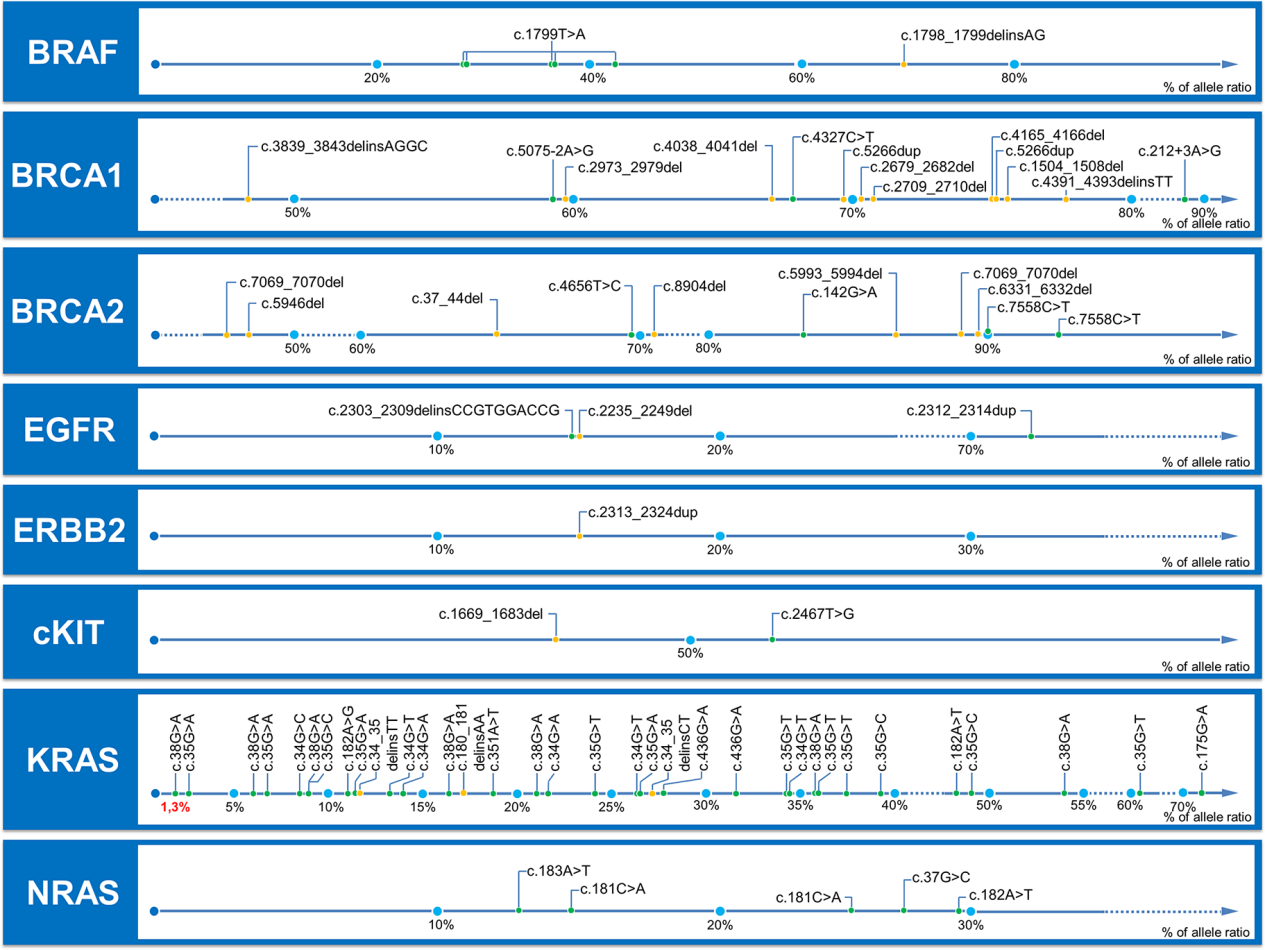
Allele-ratio of mutations tested for sensitivity evaluation Mutations are gathered by gene, and positioned along an axis which represents the observed allele ratio. SNV are represented with a green dot and indels with a yellow dot. All mutations are annotated with HGVS nomenclature. These mutations were detected previously in the time of the diagnosis by specific methods (see Materials and Methods).

**Figure 3:**
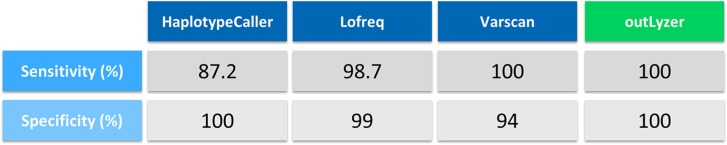
Sensitivity and specificity evaluation Sensitivity was calculated by testing previously genotyped samples harboring known mutations discovered in the time of diagnosis with contemporary validated methods which were mutation-specific (see Materials and Methods and Figure 2 for description of mutations). Specificity is calculated in KRAS codons 12 and 13 for which a sufficiently sensitive method was available (Cold-PCR followed by Pyro-sequencing). All variants detected in these specific regions by the variant-callers tested were checked for false or true positive nature.

**Figure 4:**
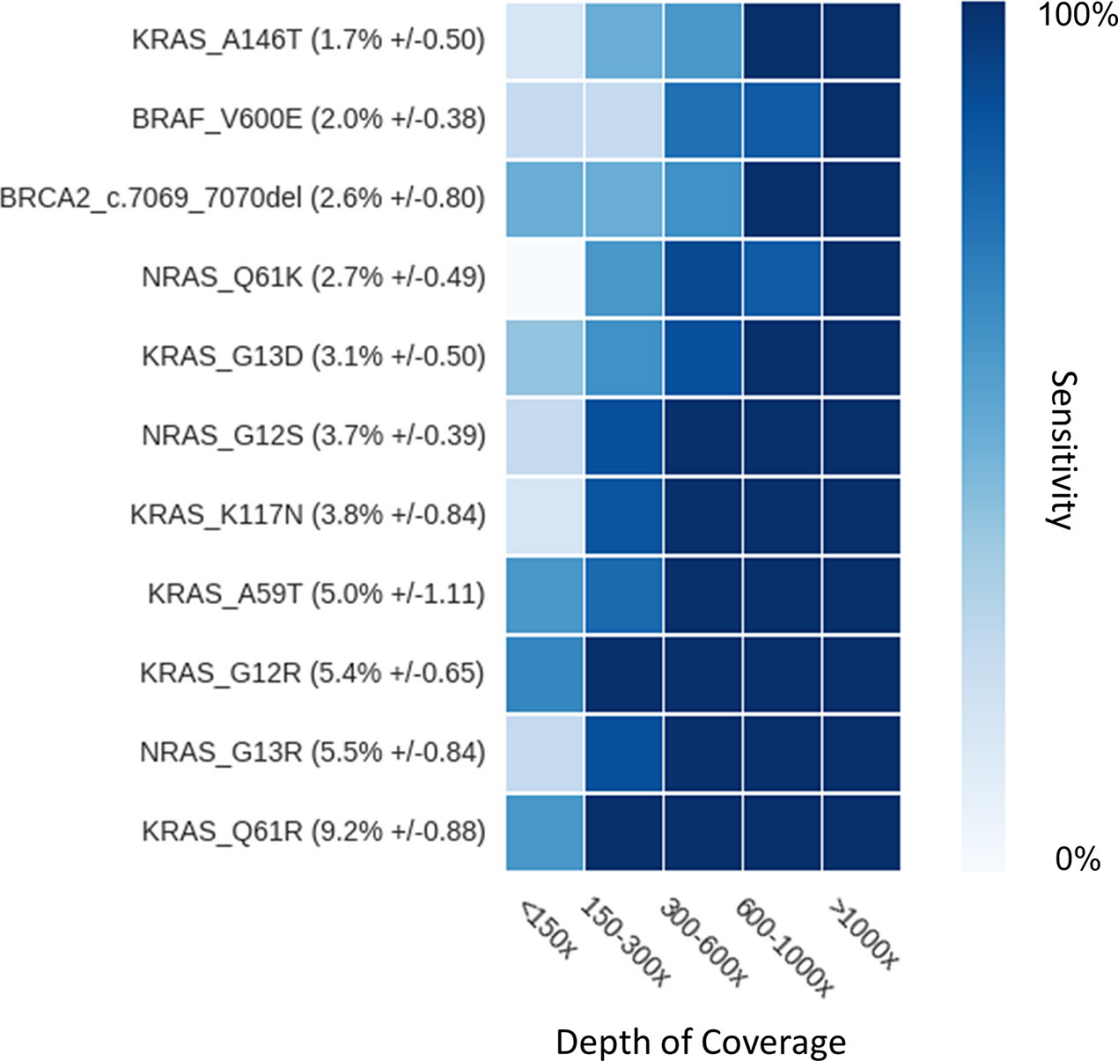
Impact of coverage on sensitivity Each mutation of interest (y-axis) is ranked according to its average allele-ratio in ascending order from top to bottom. Sensitivity is calculated for each mutation at each coverage category (x-axis), and represented by color variations from 0 to 100% as shown on color bar on the right.

Sensitivity was evaluated on all events previously genotyped, including 51 SNV and 27 indels from 1.3% to 93% of allele-ratio (Figure 2). OutLyzer had the best sensitivity with Varscan in identifying 100 % of the tested mutations, while, as awaited, HaplotypeCaller performed least well owing to a loss of sensitivity in the detection of low allele-frequency variants (Figure 2 and Figure 3). To evaluate the impact of coverage on outLyzer sensitivity, we used a sample built from the DNA of 11 tumors harbouring already known mutations. The 11 DNA were mixed in order to obtain a unique sample of DNA with 11 low allele-ratio mutations (from 1 to 10% of allele-ratio). This mixed sample was sequenced 10 times in independent experiments (reproducibility tests) and BAM files obtained were used to simulate different coverage conditions. For each BAM some “reads” were randomly selected *in silico* to divide by 2, 4 and 10 the initial depth of coverage, in order to obtain five ranges of depth of coverage on the genomic loci of the mutations (< 150×, 150–300×, 300–600×, 600–1000×, > 1000×). The sensitivity was calculated for each range of depth of coverage (Figure 4). As expected, a low coverage condition (< 150×) demonstrated a loss of sensitivity and is harmful for the detection of low allele-ratio mutations. A coverage in the range of 150–300× is sufficient to detect mutations with 5% of allele-ratio with a good sensitivity. Increasing the coverage enhance the sensitivity up to detect mutations about 1 or 2% of allele-ratio with a coverage about 1000×.

**Figure 5:**
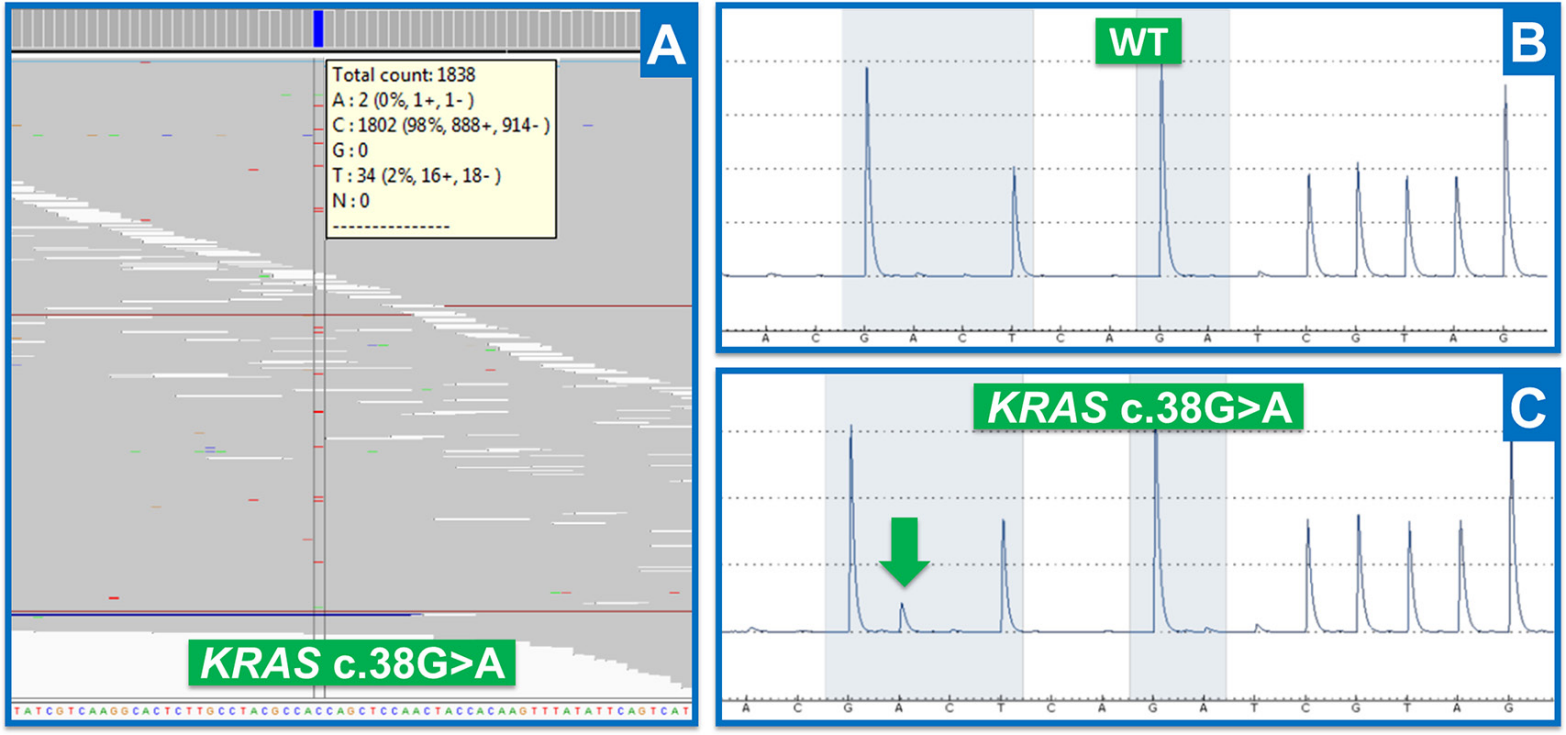
Results obtained by NGS and target-specific method (**A**) NGS results (IGV visualization): reads aligned along a reference genome, illustrating *KRAS* c.38G > A mutation for Thera41 patient (codon 13). Data are represented in genomic orientation. (**B**) Pyrosequencing results obtained for a healthy patient on *KRAS* codons 12 and 13 (Wild Type). Data are represented in transcript orientation. (**C**) Pyrosequencing results obtained for Thera41 patient (Supplementary Table S1) with *KRAS* c.38G > A mutation (green arrow). Data are represented in transcript orientation.

**Figure 6:**
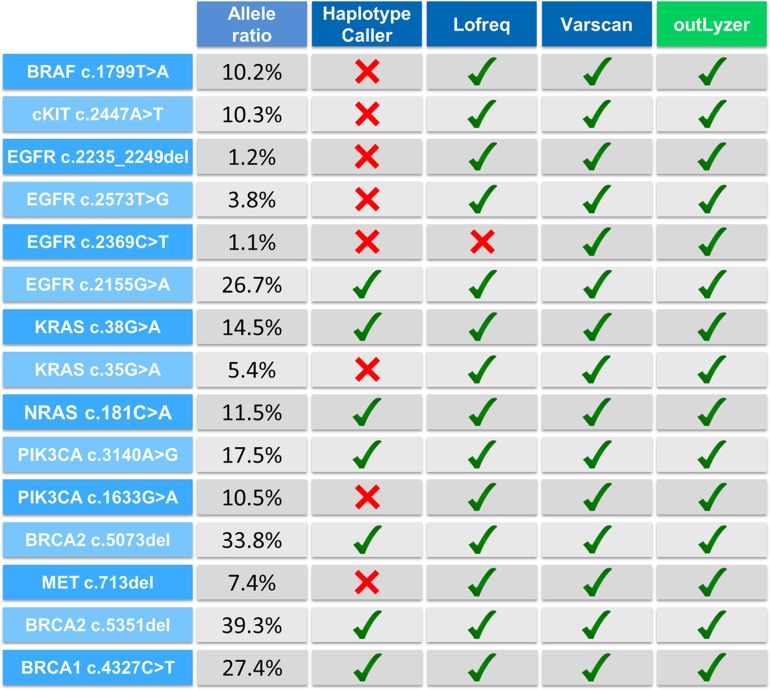
Performance of all variant-callers on HorizonDX sample Red cross means that mutation was not detected by the corresponding software.

It is difficult to test with a specific method all mutations detected by the variant-callers in order to determine their true or false positive nature, specifically regarding low allele-ratio variants. Consequently, the evaluation of the specificity was therefore restricted to the analysis of *KRAS* mutational hotspots (codon 12 and 13) for which a target specific method was already available for routine diagnostic purposes. The variant-callers detected 35 mutations in *KRAS* mutational hotspots. The corresponding samples were analyzed by Cold-PCR followed by pyrosequencing to evaluate the false or true positive nature of the mutations and then the specificity was calculated (Figure 5). OutLyzer also showed the best specificity with HaplotypeCaller by making no mistakes on the identified mutations, thereby establishing a specificity of 100% on these mutational hotspots (Figure 3). To illustrate performances of each variant-caller, a commercial sample (HorizonDX) in which a number of mutations with various allele ratios were known was sequenced and analyzed independently from other samples (Figure 6). Only OutLyzer and Varscan were able to detect all expected mutations. As awaited, HaplotypeCaller did not identify the lowest allele frequency mutations (Supplementary Table S1).

## DISCUSSION

Highlighting mutational events with a very low allele frequency is challenging but essential in oncology in the search for somatic mutations characterizing tumor heterogeneity [6]. Next Generation Sequencing (NGS) technologies have become powerful tools for helping to diagnose pathologies and establishing therapeutic strategies. However, it may be difficult to interpret their results and to differentiate false and true positives. Many tools are available to detect somatic mutations, each with its advantages and drawbacks. Tools such as HaplotypeCaller [9] were initially designed for discovering germline mutations so they are very specific but lack sensitivity, especially for detecting low-allele-frequency mutations. Other variant-callers have emerged to address this issue. Varscan [10] and Lofreq [11] had better performance here with almost all the tested mutations. However, in our hands they either generated false positive or they lose some sensitivity in case of low quality DNA samples. Others such as MuTect [12] or JointSNVMix [13], which were not tested here but have been reported to have both good sensitivity and specificity, need a paired healthy sample for mutation detection analysis. This may be problematic for the abovementioned reasons.

OutLyzer largely offsets these defects without the need for a matched healthy tissue sample by adjusting locally its sensitivity threshold depending on the sample sequencing quality, in the same way as a biologist inspects aligned sequencing data and assesses quality and background noise. This leads to better performance in terms of sensitivity and specificity and is critical in a clinical context, with all the quality constraints imposed on diagnostic laboratories nowadays. Evaluating the performance of variant-callers remains challenging, largely because of the amount of data produced by NGS technology. This revolution allows us to explore larger genomic regions with hopefully greater sensitivity. But all events discovered with this technology cannot be checked and compared systematically with conventional methods, such as Sanger sequencing, pyrosequencing or digital PCR. Therefore it appears critical to exactly know the theorical limit of each technology used. In order to understand outLyzer's limitations in its usage, in addition to variant-calling analysis, outLyzer is able to provide the analysis detection limits in the form of a minimum detectable allele ratio. This detection threshold is produced per patient, either for each region specified in an associated BED (Browser Extensible Data) File or for a specific genomic position, such as *KRAS* mutational hotspots. In a second operating mode, it is also able to provide immediately the major sequencing features for a specified genomic position, including sequencing depth, reference and alternative allele (if present), the number of forward and reverse reads that carry this alternative allele, the average sequencing quality, and an estimation of local sequencing background noise.

OutLyzer estimates the sum of several background noise sources, including sequencing mistakes, errors generated by sample preparation and bioinformatics analysis, based on an outlier detection algorithm. Here, detection of outliers is used to highlight true mutations but outlying data have to be used carefully depending on the experimentation type so as to fit the analysis as well as possible [14]. With a high depth of coverage on the diagnostic genomic regions, dedicated bioinformatics tools can help us to ensure a good sensitivity and specificity. Depending of the sensitivity required, the depth of coverage can be adapted (Figure 4), but a high sensitivity for detection of mutations with an allele-ratio of 1% requires at least 1000× of coverage for a robust analysis. Such depth of coverage can be helpful to explore tumoral heterogeneity, particularly with a low tumor cellularity, or to detect mosaic mutations or circulating tumor DNA mutations.

Despite the fact that bioinformatic, statistical and computational methods are constantly evolving, a hurdle they face is the nature of processed data, which contains errors non-distinguishable from real biological events. An important source of false positives lies in the sample preservation method, the FFPE (Formalin-Fixed, Paraffin-Embedded), which is responsible for DNA modifications considered as artifacts unrated to pathology [15]. This limitation may be not overpassed by bioinformatics improvements. Therefore, alternative sample preparation before sequencing could limit bias to enhance the detection of low allele frequency mutations. For example, sample preparation protocols based on the use of a random index can simulate the double-stranded sequencing of a unique DNA fragment with a suitable bioinformatics analysis. Such protocols could eliminate PCR and sequencing errors and distinguish true somatic mutations occurring on both strands from errors generated during the analytical process [16].

Today sequencing technologies make it possible to explore numerous diseases and characterize many genetic abnormalities. Software such as OutLyzer could prove useful by highlighting mainly true positive mutations in the sequencing background and focusing only on the most relevant information. Outlyzer sources are available on https://github.com/EtieM/outLyzer.

## MATERIALS AND METHODS

### OutLyzer implementation

OutLyzer is a tool written in Python [17] programming language that runs on Linux. It requires the SAMTOOLS [18] suite and additional python libraries: *scpiy*, *numpy*, *subprocess*, and *multiprocessing*. It was tested on Fedora 21 and CentOS 6.7 Linux distribution with SAMTOOLS 1.2 and Python 2.7.8 versions.

OutLyzer has two main operating modes: (i) as a classical variant-caller; (ii) as a tool to evaluate local quality metrics. By giving it a chromosomal for a given sample, it quickly evaluates whether a mutation is present at this position, specifies all raw sequencing information and evaluates local background noise.

OutLyzer uses BAM (Binary Alignment Map) files as input for analysis. It is preferable to use the BWA [19] / GATK [9] bioinformatic pipeline to produce BAM files, according to the Broad Institute recommendations. BAM files are converted into the pileup data format using SAMTOOLS [18]. For each genomic position of targeted regions, the number and type of alternative events on forward or reverse reads and the associated PHRED score are stored in memory for a defined genomic region to be analyzed together (ex: one exon) in the subsequent statistical steps.

For each region stored in memory, OutLyzer evaluates background noise locally by using Thompson's Tau Test [20]. This test is a statistical method for deciding whether to keep or discard a suspected outlier in a sample of a single variable. At each iteration, the sample mean $\bar{x}$ and standard deviation S are calculated. Then for each point of the sample, the absolute value of the deviation is calculated:

$$\delta_{i}=|d_{{}_{i}}|=|x_{{}_{i}}-\bar{x}|$$

The data point most suspected as a possible outlier is the data point with the maximum value of δ_i_. The value of the modified Thompson τ is calculated from the critical value of Student's t PDF (Probability Density Function):

$$\tau =\frac{t_{\alpha /2}*(n-1)}{\sqrt{n}*\sqrt{(n-2)+t_{\alpha /2}^{2}}}$$

**Figure 7:**
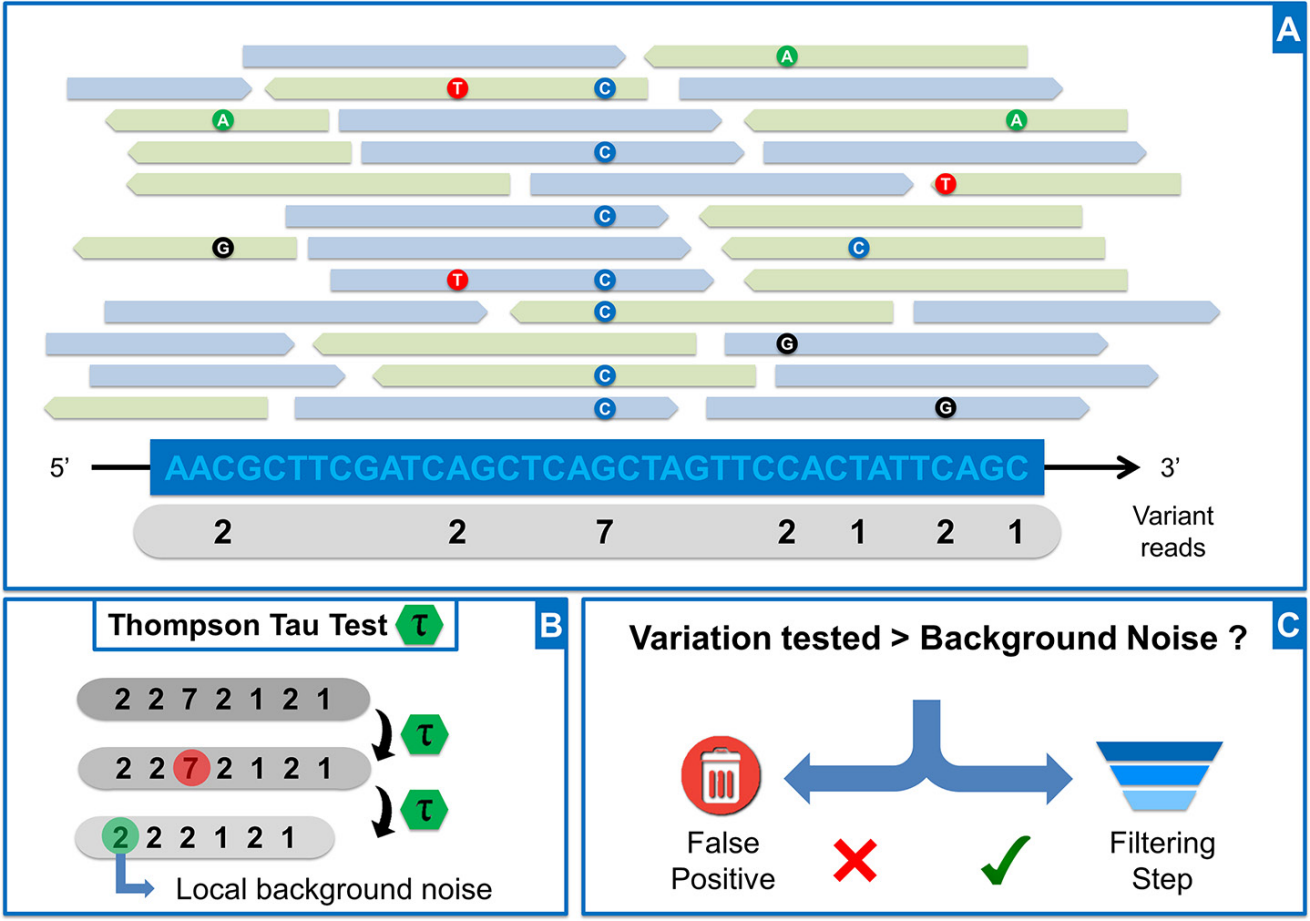
OutLyzer analysis (**A**) Representation of reads aligned along a reference genome. For each genomic position, the number of variant reads is counted and stored in a list (grey banner) (**B**) Application of Thompson Tau test on list obtained in A (**C**) The number of reads carrying the potential variant is compared to local background noise to evaluate whether the event is a false positive. If the variant is above background noise, it will pass through a filtration step based on sequencing quality, and including the reads forward-reverse balance, the average PHRED score of mutated bases, and the standard deviation of average PHRED score.

Where n is the number of data points in the sample. Student's *t* value is based on an α risk set by default at 0.001 (adjustable in settings in OutLyzer) and df (degree of freedom) = *n*–2. The removal or retention of a potential outlier is evaluated by the decision rule: if *δ_i_* > τ * S, data point is rejected from sample because considered as an outlier. Otherwise, the data point is kept. The test is performed until no outlier is found in the sample. Considering a window of 200 bp centered on potential mutations (adjustable according to user preferences), for each genomic position, the number of reads containing alternative bases is added to a list which forms a local sample (Figure 7A and 7B). First, all values equal to zero (genomic position that does not contain a mutation) are removed from the sample. Then Thompson's Tau test is performed to remove the largest outlier in the sample and the test is performed again until no outlier is found. The largest data point in the remaining list is then used to define the background noise locally (Figure 7B). Back to the aligned data, if the number of reads supporting a potential variant is higher than the local background noise previously defined, the potential variant moves to the filtration step. Otherwise it is considered as part of the background noise (Figure 7C). For each candidate variation kept in the statistical step, several filtration steps are performed, all of which are configurable: (i) variation rate must be greater than twice the background noise, (ii) variation must have an average PHRED score greater than 20, (iii) the average PHRED score must have a standard deviation below 7, (iv) the forward/reverse balance of variants should be between 30 and 70%. Variations that meet all these criteria are written in a vcf (Variant Call Format) file.

### Tumor samples

Paraffin-embedded tumor samples from 130 patients with colorectal (70 samples), ovarian (50 samples), lung (4 samples), breast (4 samples), skin (1 sample) and stomach (1 sample) cancer, were selected for high-throughput sequencing analysis (Supplementary Table S1). All patients had been previously sequenced by the target-specific techniques in the time of the initial diagnosis (Sanger sequencing, Cold-PCR followed by Pyrosequencing, SNaPSHOT).

Additionally, the Horizon DX Quantitative Multiplex Reference Standard (Horizon Discovery Group, Cambridge Research Park, Waterbeach, Cambridge, UK) was added to the dataset containing miscellaneous SNVs (Single Nucleotide Variations), insertion and deletion events at various allele frequency.

### Sequencing analysis

**Figure 8:**
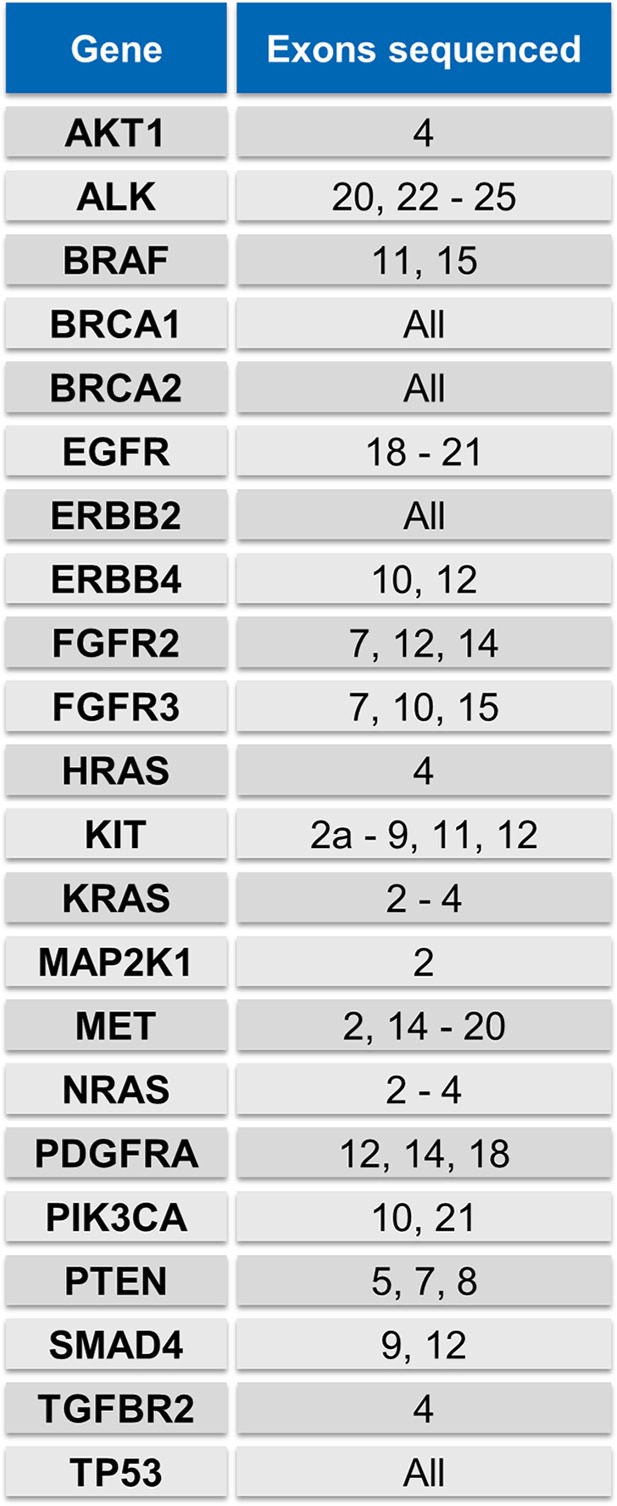
Genes included in sequencing analysis Genes have been selected to establish a NGS panel gene strategy in order to characterize solid tumors in clinical practice.

DNAs were sequenced for a panel comprised of 22 genes (Figure 8). Agilent SureDesign (Agilent, Santa Clara, CA, USA) was used to create library baits covering the exonic regions of these genes. Regions of interest were captured with the SureSelect XT Protocol (Agilent, Santa Clara, CA, USA) and sequenced on Illumina Miseq (Illumina, San Diego, CA, USA) using the paired-end 2 × 150 bp program. Bio-informatic analysis was performed with the CASAVA Suite v1.8 for demultiplexing, followed by BWA 0.7.12 for alignment and GATK v3.3 pipeline to produce BAM files, according to the Broad Institute recommendations. The variant-calling step was carried out by HaplotypeCaller, Lofreq v2.1.1 and Varscan v2.3.7 for comparison with OutLyzer (settings are described in the Supplementary information S1). Only SNVs and Indels with an allele ratio respectively greater than 1% and 2% were compared. Venn Diagram representations were designed with jVenn [21].

## SUPPLEMENTARY MATERIALS




