# Supplementary material for: OutLyzer: software for extracting low-allele-frequency tumor mutations from sequencing background noise in clinical practice
Source: Oncotarget. 2016 Nov 4;7(48):79485–93. doi: 10.18632/oncotarget.13103 (PMC5346729; doi:10.18632/oncotarget.13103)
Supplement: Supplementary file 1 [file oncotarget-07-79485-s001.pdf]

OutLyzer: software for extracting low-allele-frequency tumor mutations from sequencing background noise in clinical practice

Supplementary Materials

Supplementary Table S1: Mutations previously genotyped with target-specific techniques.

Supplementary Information S1: Commands used to run each variant caller

HaplotypeCaller: v. 3.3-0-g37228af
$ Java -jar GenomeAnalysisTK.jar -T HaplotypeCaller -R referenceGenome_hg19.fa -D dbSNP_positions.vcf -L bedFile.bed -stand_call_conf 30.0 -stand_emit_conf 10.0 -I inputBamFile.bam -o output.vcf

Lofreq: v.2.1.1
$ Lofreq call -f referenceGenome_hg19.fa --call-indels -l bedFile.bed --use-orphan -o output.vcf inputBamFile.bam

Samtools: v.1.2
Command used to generate pileup file needed by Varscan, and also directly integrated into outLyzer program.
Samtools mpileup -d 100000 -Q 0 -A -R -B -f -x -l bedFile.bed -f referenceGenome_hg19.fa inputBamFile.bam > output.mpileup

Varscan: v.2.3.7
$ java -jar Varscan.v2.3.7.jar mpileup2cns --min-var-freq 0.01 --output-vcf 1 --variants 1 inputPileupFile.mpileup > output.vcf

outLyzer : v.1.0
$ python outLyzer.py calling -core 3 -cut 3 -bed bedFile.bed -ref referenceGenome_hg19.fa -bam inputBamFile.bam -output /path/to/output/Dir/
