# Supplementary material for: OutLyzer: software for extracting low-allele-frequency tumor mutations from sequencing background noise in clinical practice
Source: Oncotarget. 2016 Nov 4;7(48):79485–93. doi: 10.18632/oncotarget.13103 (PMC5346729; doi:10.18632/oncotarget.13103)
Supplement: Supplementary file 2 [file oncotarget-07-79485-s002.pdf]

Tumor Characterization				Mutation awaiting				Allele ratio detected by corresponding variant caller			
ID	Tumor type	nor cellularity	Previously determinated molecular profile	Gene	Seq Accession	cNomen	pNomen	laplotypeCalle	Lofreq	Varscan	outLyzer
Thera01	Lung	>50%	EGFR Status known for Exons 18 - 19 - 20 - 21	EGFR	NM_005228	c.2312_2314dup	p.Asn771_Pro772insHis	72,70%	66%	67%	66,0%
Thera03	Lung	15-25%	EGFR Status known for Exons 18 - 19 - 20 - 21<br>KRAS status known for Exon 2	EGFR	NM_005228	c.2235_2249del	p.Glu746_Ala750del	17,50%	15,50%	14,82%	14,7%
Thera04	Lung	>50%	EGFR Status known for Exons 18 - 19 - 20 - 21	ERBB2	NM_004448	c.2313_2324dup	p.Tyr772_Ala775dup	36,40%	21,80%	21,87%	21,6%
Thera05	Stomach	60%		KIT	NM_000222	c.1669_1683del	p.Trp557_Glu561del	44,70%	42,80%	42,75%	42,6%
	Stomach			KIT	NM_000222	c.2467T>G	p.Tyr823Asp	61,30%	57,40%	57,41%	57,5%
Thera06	Lung	>50%	EGFR Status known for Exons 18 - 19 - 20 - 21<br>KRAS status known for Exon 2	EGFR	NM_005228	2303_2309delinsCCGTGGACG	8_Asp770delinsThrVal	17,10%	15,80%	16,00%	15,6%
Thera07	Colon	NA	RAS status known for Exon 2 - 3 - 4	NRAS	NM_002524	c.183A>T	p.Gln61His	22,20%	13,20%	13,24%	13,2%
Thera09	Skin	50%	BRAF status known for exon 15	BRAF	NM_004333	c.1798_1799delinsAG	p.Val600Arg	72,20%	68,90%	69,06%	69,0%
Thera14	Ovary	20%	Germline genotype for BRCA1 and BRCA2 known	BRCA1	NM_007294	c.3839_3843delinsAGGC	p.Ser1280X	46,50%	42,00%	47,00%	47,0%
Thera18	Ovary	NA	Germline genotype for BRCA1 and BRCA2 known	BRCA2	NM_000059	c.5946del	p.Ser1982Argfs*22	86,00%	84,10%	84,00%	83,1%
Thera20	Ovary	80%	Germline genotype for BRCA1 and BRCA2 known	BRCA1	NM_007294	c.2709_2710del	p.Cys903*	74,30%	71,20%	71,66%	70,5%
Thera22	Ovary	60%	Germline genotype for BRCA1 and BRCA2 known	BRCA1	NM_007294	c.5266dup	p.Gln1756Profs*4	79,00%	74,60%	75,72%	74,4%
Thera24	Ovary	NA	Germline genotype for BRCA1 and BRCA2 known	BRCA2	NM_000059	c.7069_7070del	p.Leu2357Valfs*2	50,80%	47,00%	47,21%	46,6%
Thera25	Ovary	70%	Germline genotype for BRCA1 and BRCA2 known	BRCA1	NM_007294	c.2679_2682del	p.Lys893Asnfs*106	76,00%	70,60%	70,82%	70,1%
Thera26	Ovary	80%	Germline genotype for BRCA1 and BRCA2 known	BRCA2	NM_000059	c.142G>A	p.Glu48Lys	84%	83,50%	83,52%	83,53%
Thera27	Ovary	70%	Germline genotype for BRCA1 and BRCA2 known	BRCA1	NM_007294	c.5266dup	p.Gln1756Profs*4	77,60%	70,40%	70,18%	69,9%
Thera29	Ovary	80%	Germline genotype for BRCA1 and BRCA2 known	BRCA2	NM_000059	c.5993_5994del	p.Gln1998Argfs*4	92,60%	93,20%	89,17%	87,3%
Thera30	Ovary	50%	Germline genotype for BRCA1 and BRCA2 known<br>KRAS status known for Exon 2	KRAS	NM_033360	c.35G>T	p.Gly12Val	24,60%	24,00%	24,05%	24,1%
Thera32	Colon	1	RAS status known for Exon 2 - 3 - 4	KRAS	NM_033360	c.38G>A	p.Gly13Asp	NA	NA	1,31%	1,3%
Thera34	Ovary	NA	Germline genotype for BRCA1 and BRCA2 known	BRCA2	NM_000059	c.5946del	p.Ser1982Argfs*22	51,70%	48,50%	47,86%	47,56%
Thera35	Ovary	20%	Germline genotype for BRCA1 and BRCA2 known	BRCA1	NM_007294	c.5075-2A>G	p.?	62,40%	58,87%	57,69%	58,9%
Thera36	Ovary	70%	Germline genotype for BRCA1 and BRCA2 known	BRCA1	NM_007294	c.4038_4041del	p.Arg1347Glufs*18	73,40%	68,12%	67,45%	67,3%
Thera41	Colon	5%	RAS status known for Exon 2 - 3 - 4	KRAS	NM_033360	c.35G>A	p.Gly12Asp	NA	1,80%	1,90%	1,9%
Thera42	Colon	10%	RAS status known for Exon 2 - 3 - 4	KRAS	NM_033360	c.35G>A	p.Gly12Asp	NA	6,80%	6,84%	6,9%
Thera43	Colon	40%	RAS status known for Exon 2 - 3 - 4	KRAS	NM_033360	c.35G>A	p.Gly12Asp	NA	11,10%	10,96%	11,2%
Thera44	Colon	20%	RAS status known for Exon 2 - 3 - 4	NRAS	NM_002524	c.181C>A	p.Gln61HLys	15%	15,10%	14,99%	15,1%
Thera45	Colon	30%	RAS status known for Exon 2 - 3 - 4	KRAS	NM_033360	c.182A>G	p.Gln61Arg	NA	11%	10,76%	11,03%
Thera51	Colon	40%	RAS status known for Exon 2 - 3 - 4	KRAS	NM_033360	c.38G>A	p.Gly13Asp	20,70%	20,90%	20,82%	20,9%
Thera52	Colon	80%	RAS status known for Exon 2 - 3 - 4	KRAS	NM_033360	c.34G>T	p.Gly12Cys	12,80%	13,60%	13,68%	13,6%
Thera54	Colon	10%	RAS status known for Exon 2 - 3 - 4	KRAS	NM_033360	c.351A>T	p.Lys117Asn	20%	18,70%	18,82%	18,8%
Thera55	Colon	20%	RAS status known for Exon 2 - 3 - 4	KRAS	NM_033360	c.436G>A	p.Ala146Thr	32,60%	31,80%	32,07%	31,8%
Thera56	Colon	20%	RAS status known for Exon 2 - 3 - 4	KRAS	NM_033360	c.35G>C	p.Gly12Ala	NA	9,30%	9,23%	9,3%
Thera58	Colon	40%	RAS status known for Exon 2 - 3 - 4	KRAS	NM_033360	c.34G>C	p.Gly12Arg	NA	9,06%	8,92%	9,1%
Thera60	Colon	30%	RAS status known for Exon 2 - 3 - 4	NRAS	NM_002524	c.37G>C	p.Gly13Arg	28%	27,30%	27,26%	27,4%
Thera61	Colon	10%	RAS status known for Exon 2 - 3 - 4	NRAS	NM_002524	c.181C>A	p.Gln61Lys	29,70%	25,40%	25,26%	25,4%
Thera65	Ovary	60%	Germline genotype for BRCA1 and BRCA2 known	BRCA2	NM_000059	c.4656T>C	p.=	72,12%	69,83%	70,04%	69,8%
Thera69	Ovary	60%	Germline genotype for BRCA1 and BRCA2 known	BRCA1	NM_007294	c.4327C>T	p.Arg1443*	69,70%	68,33%	68,50%	68,3%
Thera75	Colon	30%	RAS status known for Exon 2 - 3 - 4	KRAS	NM_033360	c.34_35delinsCT	p.Gly12Leu	27,08%	27,20%	26,70%	27,2%
Thera76	Colon	NA	RAS status known for Exon 2 - 3 - 4	KRAS	NM_033360	c.180_181delinsAA	p.Gln61Lys	15,70%	16,70%	16,90%	16,7%
Thera78	Ovary	70%	Germline genotype for BRCA1 and BRCA2 known	BRCA1	NM_007294	c.4165_4166del	p.Ser1389X	78,30%	74,74%	74,65	74,3%
Thera79	Ovary	70%	Germline genotype for BRCA1 and BRCA2 known	BRCA2	NM_000059	c.7558C>T	p.Arg2520X	90,80%	89,95%	90,10%	90,0%
Thera80	Ovary	NA	Germline genotype for BRCA1 and BRCA2 known	BRCA2	NM_000059	c.6331_6332del	p.Lys2111GlufsX17	94,20%	91,90%	90,12%	89,8%

Thera81	Ovary	80%	Germline genotype for BRCA1 and BRCA2 known	BRCA2	NM_000059	c.8904del	p.Val2969CysfsX7	71%	71,56%	71,19%	70,7%
Thera83	Ovary	70%	Germline genotype for BRCA1 and BRCA2 known	BRCA1	NM_007294	c.1504_1508del	p.Leu502AlafsX2	87,20%	75,90%	75,96%	75,4%
Thera84	Ovary	80%	Germline genotype for BRCA1 and BRCA2 known	BRCA1	NM_007294	c.212+3A>G	p.?	87,80%	88,96%	89,07%	89,0%
Thera86	Ovary	80%	Germline genotype for BRCA1 and BRCA2 known	BRCA2	NM_000059	c.37_44del	p.Glu13X	82,60%	65,96%	65,17%	64,8%
Thera87	Ovary	60%	Germline genotype for BRCA1 and BRCA2 known	BRCA2	NM_000059	c.7558C>T	p.Arg2520X	94,50%	93,50%	93,56%	93,5%
Thera88	Ovary	80%	Germline genotype for BRCA1 and BRCA2 known	BRCA1	NM_007294	c.4391_4393delinsTT	p.Pro1464LeufsX2	78,50%	77,47%	77,60%	77,5%
Thera91	Ovary	50%	Germline genotype for BRCA1 and BRCA2 known	BRCA2	NM_000059	c.7069_7070del	p.Leu2357ValfsX2	90,50%	89,72%	89,24%	89,1%
Thera94	Ovary	30%	Germline genotype for BRCA1 and BRCA2 known	BRCA2	NM_000059	c.7495C>T	p.Gln2499*	45,50%	45,80%	45,80%	45,8%
Thera99	Ovary	NA	Germline genotype for BRCA1 and BRCA2 known	BRCA1	NM_007294	c.2973_2979del	p.Lys991Asnfs*7	65,40%	60,10%	60,15%	59,7%
Thera123	Colon	20%	RAS status known for Exon 2 - 3 - 4	KRAS	NM_033360	c.38G>A	p.Gly13Asp	NA	5,90%	5,64%	6,0%
Thera124	Colon	50%	RAS status known for Exon 2 - 3 - 4	KRAS	NM_033360	c.38G>A	p.Gly13Asp	16,60%	16,40%	16,63%	16,4%
Thera126	Colon	60%	RAS status known for Exon 2 - 3 - 4	KRAS	NM_033360	c.175G>A	p.Ala59Thr	73,90%	71,40%	71,27%	71,4%
Thera127	Colon	40%	RAS status known for Exon 2 - 3 - 4	KRAS	NM_033360	c.35G>A	p.Gly12Asp	28,27%	26,67%	26,32%	26,7%
Thera128	Colon	30%	BRAF status known for Exon 15	BRAF	NM_004333	c.1799T>A	p.Val600Glu	37,31%	36,38%	36,04%	36,4%
Thera132	Colon	50%	RAS status known for Exon 2 - 3 - 4	KRAS	NM_033360	c.35G>C	p.Gly12Ala	39,87%	39,60%	39,72%	39,6%
Thera138	Colon	40%	RAS status known for Exon 2 - 3 - 4	KRAS	NM_033360	c.38G>A	p.Gly13Asp	54,70%	54,33%	54,73%	54,3%
Thera140	Colon	60%	RAS status known for Exon 2 - 3 - 4	KRAS	NM_033360	c.35G>T	p.Gly12Val	38,00%	37,30%	37,84%	37,3%
Thera146	Colon	50%	RAS status known for Exon 2 - 3 - 4	KRAS	NM_033360	c.436G>A	p.Ala146Thr	25,90%	27,70%	27,69%	27,7%
Thera148	Colon	45%	BRAF status known for Exon 15	BRAF	NM_004333	c.1799T>A	p.Val600Glu	26,80%	28,60%	28,16%	28,6%
Thera 151	Colon	30%	RAS status known for Exon 2 - 3 - 4	KRAS	NM_033360	c.38G>A	p.Gly13Asp	NA	9,30%	9,42%	9,3%
Thera 152	Colon	20%	RAS status known for Exon 2 - 3 - 4	KRAS	NM_033360	c.34G>A	p.Gly12Ser	22,30%	21,40%	21,25%	21,4%
Thera 153	Colon	10%	RAS status known for Exon 2 - 3 - 4	KRAS	NM_033360	c.182A>T	p.Gln61Lys	51%	47,80%	48,13%	47,8%
Thera 155	Colon	40%	RAS status known for Exon 2 - 3 - 4	KRAS	NM_033360	c.35G>C	p.Gly12Ala	48%	48,80%	48,92%	48,8%
Thera 157	Colon	30%	BRAF status known for Exon 15	BRAF	NM_004333	c.1799T>A	p.Val600Glu	28,70%	28,50%	28,37%	28,6%
Thera 158	Colon	20%	RAS status known for Exon 2 - 3 - 4	KRAS	NM_033360	c.35G>T	p.Gly12Val	35,20%	35,70%	35,70%	35,7%
Thera 159	Colon	40%	BRAF status known for Exon 15	BRAF	NM_004333	c.1799T>A	p.Val600Glu	43,80%	42%	42%	42,1%
Thera 160	Colon	20%	RAS status known for Exon 2 - 3 - 4	KRAS	NM_033360	c.34G>A	p.Gly12Ser	14,30%	13,90%	14,08%	14,0%
Thera 162	Colon	40%	RAS status known for Exon 2 - 3 - 4	KRAS	NM_033360	c.34G>T	p.Gly12Cys	25,40%	26,60%	26,76%	26,6%
Thera 163	Colon	80%	RAS status known for Exon 2 - 3 - 4	KRAS	NM_033360	c.35G>T	p.Gly12Val	63,80%	60%	60%	60,1%
Thera173	Colon	70%	RAS status known for Exon 2 - 3 - 4	KRAS	NM_033360	c.35G>T	p.Gly12Val	36,50%	34,60%	34,49%	34,6%
Thera174	Colon	80%	RAS status known for Exon 2 - 3 - 4	NRAS	NM_002524	c.182A>T	p.Gln61Lys	28%	29,40%	29,75%	29,5%
Thera175	Colon	50%	RAS status known for Exon 2 - 3 - 4	KRAS	NM_033360	c.34G>T	p.Gly12Cys	35,70%	34,70%	34,56%	34,7%
Thera176	Colon	50%	RAS status known for Exon 2 - 3 - 4	KRAS	NM_033360	c.38G>A	p.Gly13Asp	34,90%	35,60%	35,81%	35,6%
Thera178	Colon	30%	BRAF status known for Exon 15	BRAF	NM_004333	c.1799T>A	p.Val600Glu	29,60%	28,70%	28,43%	28,8%
Thera179	Colon	50%	BRAF status known for Exon 15	BRAF	NM_004333	c.1799T>A	p.Val600Glu	36,30%	36,30%	36,31%	36,3%
Thera182	Colon	NA	RAS status known for Exon 2 - 3 - 4	KRAS	NM_033360	c.34_35delinsTT	p.Gly12Phe	NA	12,30%	12,32%	12,3%
